# Development of a rapid screen for the endodermal differentiation potential of human pluripotent stem cell lines

**DOI:** 10.1038/srep37178

**Published:** 2016-11-22

**Authors:** Richard Siller, Elena Naumovska, Santosh Mathapati, Max Lycke, Sebastian Greenhough, Gareth J. Sullivan

**Affiliations:** 1 Department of Molecular Medicine, Institute of Basic Medical Sciences, Faculty of Medicine, University of Oslo, PO Box 1112 Blindern, 0317 Oslo, Norway; 2 Norwegian Center for Stem Cell Research, PO Box 1112 Blindern, 0317 Oslo, Norway; 3 Institute of Immunology, Oslo University Hospital-Rikshospitalet, PO Box 4950 Nydalen, Oslo 0424, Norway

## Abstract

A challenge facing the human pluripotent stem cell (hPSC) field is the variability observed in differentiation potential of hPSCs. Variability can lead to time consuming and costly optimisation to yield the cell type of interest. This is especially relevant for the differentiation of hPSCs towards the endodermal lineages. Endodermal cells have the potential to yield promising new knowledge and therapies for diseases affecting multiple organ systems, including lung, thymus, intestine, pancreas and liver, as well as applications in regenerative medicine and toxicology. Providing a means to rapidly, cheaply and efficiently assess the differentiation potential of multiple hPSCs is of great interest. To this end, we have developed a rapid small molecule based screen to assess the endodermal potential (EP) of hPSCs, based solely on definitive endoderm (DE) morphology. This drastically reduces the cost and time to identify lines suitable for use in deriving endodermal lineages. We demonstrate the efficacy of this screen using 10 different hPSCs, including 4 human embryonic stem cell lines (hESCs) and 6 human induced pluripotent stem cell lines (hiPSCs). The screen clearly revealed lines amenable to endodermal differentiation, and only lines that passed our morphological assessment were capable of further differentiation to hepatocyte like cells (HLCs).

During development, the process of lineage specification causes the totipotent zygote to undergo a series of differentiation steps during which the three embryonic germ layers are specified: ectoderm, mesoderm and endoderm. The endodermal lineage is the germ layer which contributes to a number of critical organs including the thymus, lungs, liver, pancreas, and intestines^1^. The endodermal lineage is specified through a number of signaling pathways during embryonic development, notably WNT/B-Catenin, Activin/NODAL and BMP signaling^1^,^2^. In order to coax human pluripotent stem cells (hPSCs) to form definitive endoderm (DE) *in vitro*, many groups have developed procedures to mimic *in vivo* embryonic conditions. To this end, a number of protocols have been developed that employ growth factors and small molecules to activate pathways in a developmentally relevant order^1^,^3^,^4^,^5^,^6^,^7^,^8^,^9^. To date, the majority of protocols rely on the use of Activin A to drive endodermal differentiation, and indeed it has been thought that Activin A was essential for *in vitro* endodermal differentiation^2^. However, a number of studies have recently shown that WNT signaling is also critical for the initiation of differentiation^2^, as well as the maintenance of the DE marker sex determining region y-box 17 (SOX17)^10^, and indeed our recent publication has proven that activation of the WNT pathway alone can efficiently differentiate hPSCs to DE^11^.

hPSCs hold great potential in fields as diverse as disease modeling, toxicity screening, cellular therapy and regenerative medicine (See Review Siller *et al*. 2013^12^). Additionally, hiPSCs, derived from patients can be utilised to interrogate disease mechanisms and provide a potential source of autologous cells for regenerative medicine^13^. As a consequence, researchers have heavily invested in developing defined protocols enabling the derivation of terminally differentiated progeny representing various tissue types. Much progress has been made in this aspect of stem cell research, especially for the neuronal lineage. This is exemplified by the initiation of clinical trials with human embryonic stem cell (hESC) derived retinal pigmented epithelium^14^,^15^. To date a plethora of hESC lines and more recently hiPSC lines have been derived in order to dissect disease^13^. Another ongoing effort is the establishment of biobanks with sufficient genetic diversity to provide human leukocyte antigen (HLA) coverage for entire populations^16^. However, a critical challenge to overcome with all these efforts will be to effectively assess these resources with respect to their ability to differentiate into the desired cell types of interest. This is highlighted by mounting evidence of hESC line variability and clonal differences observed in hiPSCs with respect to their differentiation propensities^17^,^18^,^19^,^20^,^21^,^22^,^23^ (for a detailed review see Cahan and Daley 2013^24^). With respect to the endodermal lineage, the Yamanaka group observed that hiPSC lines derived from a number of different donors demonstrated a high degree of variability in terms of their capacity to differentiate to hepatocytes^25^. This highlights the necessity for efficient, unambiguous, cost effective and rapid methods to assess if a line is up to the job, with respect to differentiation potential.

There are many reports that describe the *in vitro* production of endodermal cell types from hPSCs: thymic epithelial cells^26^,^27^,^28^, pancreatic beta cells^29^, lung epithelial cells^30^, intestinal cells^31^, cholangiocytes^32^,^33^,^34^,^35^ and hepatocytes^3^,^5^,^6^,^11^,^36^,^37^,^38^. We recently developed an efficient, small molecule driven method to direct hPSCs to hepatocyte like cells (HLCs)^12^. This novel small molecule driven approach is divided into three distinct phases which mirror the predicted developmental pathway from hPSCs to HLCs: Phase I directs the hPSCs towards DE; Phase II then drives hepatic progenitor specification; and finally Phase III generates HLCs. The small molecule derived HLCs (smHLCs) display key hepatic attributes such as serum protein production and Cytochrome P450 activity to name a few. The smHLCs are functionally equivalent to published growth factor based methodologies and importantly can be produced at a greatly reduced cost and variability when compared with traditional growth factor driven approaches.

During the differentiation process we observed dramatic morphological changes over the two days of the procedure (DE induction; Phase I) (See Fig. 1). After the first day the colonies change from a typical flat hPSC morphology, were one observes high nuclear to cytoplasmic ratio to domed, bright 3D colonies with no evidence of any cellular migration. However, by the end of second day, there has been extensive cellular migration and proliferation, with the cells taking on a typical petal/cobblestone like morphology. These observed morphological changes are concomitant with dramatic transcriptional change, including the rapid induction of *NODAL* within 4 hours of administration of CHIR99021, demonstrating a transition towards Primitive Streak (PS). This was rapidly followed by the upregulation of the PS marks brachyury (*T*), mix paired-like homeobox (*MIXL1*) and goosecoid (*GSC*) (See Fig. 1). By 48 hours, the DE markers forkhead box protein (*FOXA2*), *GSC, SOX17*, hematopoietically expressed homeobox (*HHEX*), and cerberus (*CER1*) were robustly expressed. With this in mind, we sought to address the issue of cell line variability with respect to endodermal differentiation potential (EP). We utilised a component of our novel small molecule based protocol to develop a method that leverages off these profound morphological changes in order to determine the EP of multiple hPSC lines (see Supplementary Fig. S1). To this end, we interrogated multiple hPSC lines *via* a simple and scalable small molecule based approach. In all, 10 lines were assessed for their EP. Of these 10 lines, 9 were found to be amenable to endodermal differentiation, while 1 was not. After the initial screen, we further assessed 4 lines ability to undergo differentiation to smHLCs, 3 of which had passed the screen and 1 that had not. As predicted, only the 3 lines identified to be competent for endoderm potential, were able to progress to smHLCs. Here we report a simple, robust, cost effective and rapid screen capable of assessing multiple hPSC lines for their EP purely by morphology.

## Results

### Optimisation of EP Screen using the hESC line H1

We initially assessed the EP of the hESC line H1 (WiCell)^39^, as this line in our hands was shown to be highly amenable to differentiation to HLCs^11^. To assess the EP of this line, we tested a number of differentiation regimes using Roswell Park Memorial Institute medium (RPMI)/B27 as a base media. It has been reported that insulin/PI3K signaling can be inhibitory with respect to the efficient production of DE^8^. It has been demonstrated that small molecules such as the PI3 kinase inhibitor LY294002 effectively inhibited insulin signaling^8^, whilst Sekine and colleagues (2012)^7^ demonstrated that the simple omission of insulin efficiently generated DE. Therefore we assessed B27 supplemented either with insulin or without. Another parameter that was investigated was the concentration of the GSK3 beta inhibitor CHIR99021. We previously performed a concentration assessment for CHIR99021 (1–12 μM) and identified that the optimal conditions spanned 3–4 μM range (data not shown). Therefore for all experimental screens outlined below we used the following 4 conditions: RPMI/B27 +/− insulin with either 3 or 4 μM CHIR99021 (see Supplementary Fig. S1).

On initiation of the screen the cells underwent profound morphological changes over the two day period. On day 1, the cells had transitioned from a typical flat hPSC colony morphology to bright almost 3 dimensional (3D) colonies (see Fig. 2). There was very little evidence of cell migration out of these bright colonies at day 1. By day 2 of the screen, the cells had proliferated with cells clearly migrating out from the colonies, filling the space that was present on day 1. These cells had a classic petal/cobblestone morphology which is indicative of DE^1^. In the case of the hESC line H1, all 4 DE induction regimes yielded efficient differentiation. Repeated experiments demonstrated that the most reliable and robust differentiation was achieved using RPMI supplemented with B27 containing insulin, and supplemented with 3 μM CHIR99021 (see Fig. 2 – indicated by *). In order to validate these morphology-based findings, we assessed the cells at day 1 and day 2 using a battery of developmentally relevant markers *via* RT-qPCR. On day 1 of endodermal differentiation the cells are expected to proceed to PS, and we therefore assessed key PS markers and observed significant upregulation of *NODAL, FOXA2, GSC, MIXL1* and *T* (Fig. 3a). By day 2 of the differentiation the cells should have transited through PS to DE, in accordance we observed a down-regulation of *MIXL1, GSC, NODAL*, and *T* levels, while markers of DE namely, *FOXA2, SOX17, CER1, HHEX* and *GATA4* were significantly upregulated (see Fig. 3a). To further corroborate these findings, we performed immunohistochemistry to assess the expression of SOX17 and FOXA2 in the putative DE populations, we observed strong staining for both proteins with the most robust staining observed when using RPMI supplemented with B27 with insulin and 3 μM CHIR99021 (see Fig. 3b).

### Application of EP Screen to Multiple hPSC lines

In order to assess if the EP screen was a generic approach to assess the ability of hPSCs to generate endoderm, we screened an additional 9 hPSC lines. Figure 4 and Supplementary Figs S2 and S3 illustrate the endpoint morphology of the cells under each of the 4 conditions described above. We performed the EP screen on the hiPSC lines Det RA, Det RB, Det RC, BJ S1, CRL R5, CRL S23 and the hESC lines 207, 360 and 429. These all demonstrated efficient DE production with 4 μM CHIR99021 supplemented RPMI/B27 without insulin, with the exception of CRL S23 which did not demonstrate any endodermal differentiation capacity in any of these conditions (see Fig. 4 and Supplementary Figs S2 and S3). Interestingly, the presence of insulin appeared to be refractory for DE production resulting in very limited differentiation for all these cell lines (see Fig. 4 and Supplementary Figs S2 and S3). Importantly, the morphological differences between the conditions were clear, with successful differentiations demonstrating profound cell migration out of the colonies and petal like cells in abundance. The unsuccessful differentiations were marked by tight, compact cells, which had not migrated out of the colonies.

To confirm the morphological analysis of the EP screen, cells were collected at the termination of the screen (day 2) and a number of DE markers were analysed using RT-qPCR: *FOXA2, SOX17, HHEX* and *CER1* (see Fig. 5, and Supplementary Fig. S4). In all cases the highest levels of induction were observed when the hPSCs were treated with 4 μM CHIR99021 supplemented RPMI/B27 without insulin. As expected, we observed limited expression of DE genes in the hiPSC line CRL S23 under all conditions, confirmation that this line was recalcitrant to differentiation to DE.

Again to further corroborate the data we performed further verification using immunohistochemistry against FOXA2 and SOX17 (see Fig. 6 and Supplementary Figs S5, S6, and S8). The hPSCs lines that demonstrated robust DE morphology at day 2 (RPMI/B27 minus insulin supplemented with 4 μM CHIR99021) presented with strong nuclear staining for FOXA2 and SOX17. Importantly, the hiPSC line CRL S23 that did not pass the EP screen, i.e. did not present with DE morphology, had minimal FOXA2 and SOX17 staining evident. In order to further validate the observed morphological data to the efficiency of DE differentiation, we performed quantitative immunohistochemistry against SOX17 (see Supplementary Fig. S8). This clearly demonstrated that the optimal conditions identified in the EP screen for each line correlated to the highest percentage of SOX17 positive cells at day 2. Thus, expression and staining of key endodermal markers correlate to the observed morphological characteristics, confirming the utility of a morphological based screen.

### Validation of EP Screen by HLC Differentiation

To further validate the true utility of the above EP screen, we used 4 of the above hPSC lines and assessed their ability to differentiate to HLCs. We used 3 hPSCs that had passed the initial EP screen (hESC lines H1 and 207; hiPSC line Det RA) and 1 hPSC line that appeared recalcitrant to DE production (CRL S23). We utilised a small molecule approach to differentiate the 4 hPSCs to HLCs as previously described by Siller and colleagues (2015)^11^. Each line was initially differentiated to DE under the 4 conditions used in the initial EP screen, and after DE induction the cells were then differentiated to smHLCs^11^. At day 17 the cells were assessed by phase contrast microscopy, for the presence of smHLCs (see Fig. 7). Typical HLC morphology was deemed present when the cells presented with bright junctions, and a polygonal shape. HLCs were observed for the following lines, H1, Det RA and 207 (see Fig. 7). However, the hiPSC line CRL S23 did not present with HLC morphology under any of the conditions used, as was observed for the other lines. In agreement with the initial EP screen, the most robust HLC morphology was observed for the hESC line H1 when differentiated using 3 μM CHIR99021 in the presence of insulin (see Fig. 7). For the hPSC lines 207 and Det RA the best morphology was observed when using 4 μM CHIR99021 without insulin in concordance with the EP screen (Fig. 4). In agreement with these results, the best hepatic morphology was observed when using these conditions (Fig. 7). However, CRL S23 did not show any evidence of HLC morphology under any of the tested DE induction conditions (Fig. 7), which was in accordance with the initial EP screen.

### Functional Assessment of HLCs

To further confirm the observed hepatic identity in the 3 lines (H1, Det RA and 207) and the lack of HLCs in CRL S23, we performed a battery of functional assays to verify their hepatic identity. We assessed cytochrome P450 3A4 (CYP3A4) activity in the derived smHLCs (under all conditions), by analysing the ability of the cells to induce CYP3A4 upon challenge with the proto-typical inducer Rifampicin. In agreement with the morphological assessment the lines H1, 207 and Det RA demonstrated the optimal inducibility when using the ideal conditions identified in the initial EP screen (Fig. 8a). The CRL S23 line did not display any significant CYP3A4 activity/inducibility upon challenge with Rifampicin and indeed had much lower basal CYP3A4 activity levels compared with the other successful lines (Fig. 8a).

In addition to CYP3A4 activity, Albumin production and secretion was assessed using ELISAs. Figure 8b demonstrates that as expected the ideal DE induction conditions that were identified in the EP screen correlated with the highest levels of Albumin production. Furthermore, the CRL S23 did not show any detectable Albumin production, thus indicating that the cells had not differentiated to a hepatic fate.

We additionally assessed the differentiated HLCs for their ability to synthesis and store glycogen, *via* Periodic-Acid Schiff (PAS) staining. Supplementary Fig. S7 unequivocally confirms that the EP screen was able to correctly identify the ideal differentiation conditions, and distinguish lines amenable to endodermal differentiation from those which were not capable of forming endoderm.

## Discussion

The technology of cellular reprogramming has led to the generation of thousands of disease-specific lines, as well as normal healthy controls. These can in turn can be used in many applications including disease modeling and regenerative medicine^12^. However, a critical challenge that faces the hPSC field is the issue of cell line variability with respect to differentiation potential. Initially this inherent variability was attributed to differences in the epigenetic silencing of the transgenes which were used to reprogramme the somatic cells^40^. Further studies have however shown that genetic aberrations present in hiPSC clones are not *de novo* mutations generated during the reprogramming process, but are most likely derived from the original somatic cells from which the hiPSC were reprogrammed^41^. However, further studies have also implicated culture conditions used for the maintenance of hPSCs as being one of the major drivers of inter-line variability both between hiPSC lines as well as hESC^42^,^43^. A number of publications, such as Bock (2011), have demonstrated that by analysing the transcriptional and epigenetic profile of multiple hPSC lines, it is possible to predict individual heterogeneity in differentiation potential and thus choose which lines are best suited for further differentiation to a cell type of interest^42^. The drawback of this type of approach is primarily in the high cost and intense data analysis that is required to predict the lines’ differentiation potential. Furthermore, the prediction of differentiation potential must be further assessed *in vitro*, leading to increased time and cost before each line can be fully characterised. Therefore it would be advantageous for the stem cell field to identify robust, rapid screening techniques that allow for unambiguous determination of the differentiation potential of multiple pluripotent stem cell lines at a relatively low cost.

In the case of endoderm field, there have been a number of approaches developed to screen hPSC lines for endodermal potential. Jiang and colleagues described that endogenous expression levels of *WNT3* in hESCs was a biomarker capable of predicting which hPSC lines were most amenable to endoderm differentiation^44^. While this was a useful observation and allowed many lines to be assessed by RT-qPCR, it still required validation by further *in vitro* differentiation. Other groups, such as Hay and colleagues developed an embryoid body (EB) based approach in which the undirected endodermal differentiation potential of hPSC lines is assessed through analysis of AFP upon EB maturation^45^. While this approach assesses the unguided differentiation potential of hPSC lines, it is subject to variation due to heterogeneity in EB size and quality. Additionally, this approach requires a relatively long period of differentiation and maturation of the EBs, as well as analysis through the use of immunohistochemistry, which further increases the cost of assessing a large number of hPSC lines.

**Figure 1:**
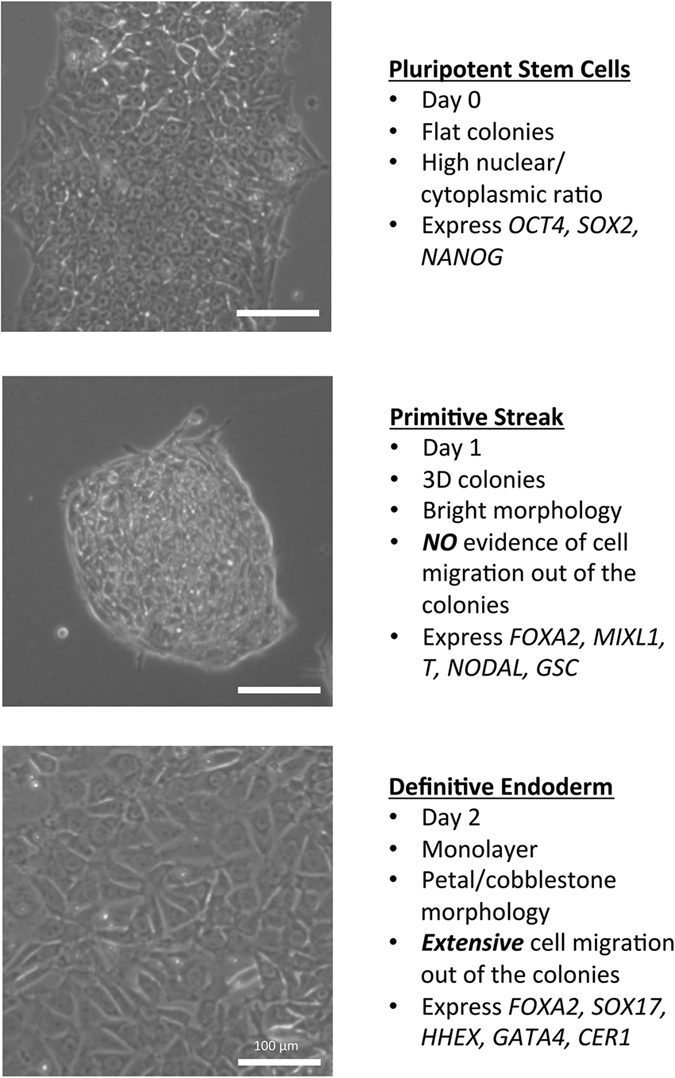
EP screen schematic. Morphological changes observed during the differentiation of human pluripotent stem cells (hPSC) through primitive streak (PS) to definitive endoderm (DE). The top panel shows a typical example of a hPSC colony grown under feeder free conditions, exhibiting typical flat colonies, a tight edge, and a high nuclear to cytoplasmic ratio, with prominent nucleoli. The middle panel shows the typical morphology after 24 hours of initiation of differentiation (PS). The colonies are domed in appearance (3 dimensional), and appear bright and compact by microscopy. Importantly, there is no evidence of cellular migration out from theses colonies. The bottom panel shows the typical morphology expected at the DE stage. There has been extensive migration out from the original colonies and the resulting DE exhibits a petal or cobblestone like morphology. The column to the right of the images describes the morphological and gene expression characteristics of the cells at each stage of the differentiation. Scale bar 100 μM. See also Supplementary Fig. S1.

**Figure 2:**
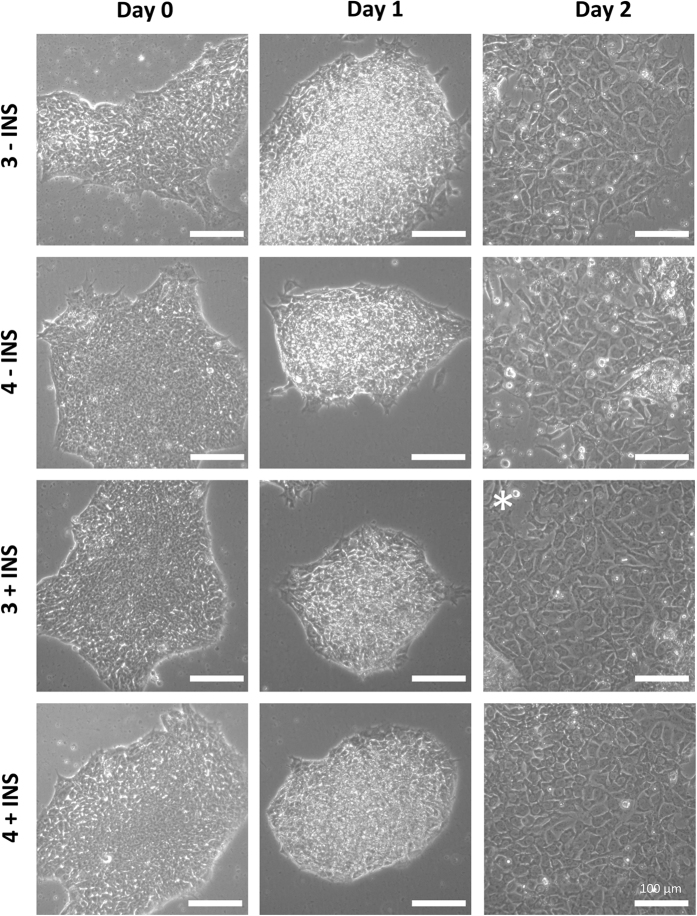
Human embryonic stem cell line H1 EP screen morphology. Endodermal Potential Screen performed on hESC line H1. H1 hESCs were assessed for their potential to produce definitive endoderm (DE) using 4 different conditions: 3 or 4 μM CHIR99021 in the absence of Insulin (indicated on the left as either 3 or 4 −INS); 3 or 4 μM CHIR99021 in the presence of Insulin (indicated on the left as either 3 or 4 +INS). The morphological changes over the 2 day differentiation period were observed and photographed using phase contrast microscopy. The white asterisk indicates the optimal condition. Scale bar 100 μM.

**Figure 3:**
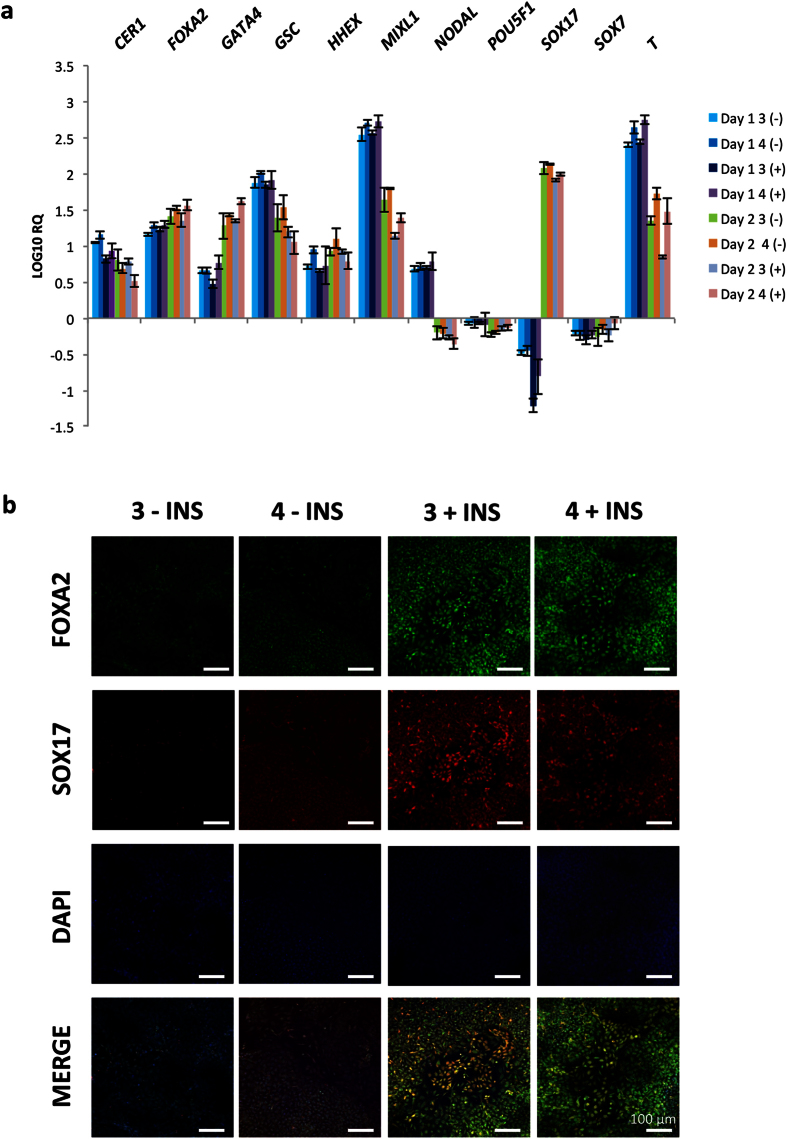
Human embryonic stem cell line H1 EP screen validation: Gene expression and immunohistochemistry. (**a**) Gene expression changes were measured using TaqMan pRT-PCR at 24 hour (Day 1) and 48 hour (Day 2) time points for the four conditions 3 or 4 μM CHIR99021 in RPMI supplemented with either B27 +/− insulin. The following genes were analysed: Pluripotency: POU class 5 homeobox 1 (POU5F1); Primitive Streak: brachyury (T), NODAL, mix paired-like homeobox 1 (MIXL1), goosecoid (GSC), forkhead box a2 (FOXA2), and cerberusrus 1 (CER1); Definitive Endoderm: CER1, FOXA2, GATA binding protein 4 (GATA4), hematopoietically expressed homeobox (HHEX), and SRY (sex determining region Y)-box 17 (SOX17); and Primitive/Extra-embryonic Endoderm: SRY (sex determining region Y)-box 7 (SOX7). The Y-axis represents the LOG10 relative quantification (RQ). All samples were normalized to beta-actin (ACTB), and to undifferentiated hESC H1 cells. Data is presented as the average of three independent experiments +/− the standard deviation. (**b**) Assessment of expression of FOXA2 (green) and SOX17 (red) by immunohistochemistry at the endpoint (48 hours/day 2) of the EP screen, imaged using confocal microscopy. Differentiation conditions are indicated at the head of each column. Scale bar 100 μM.

**Figure 4:**
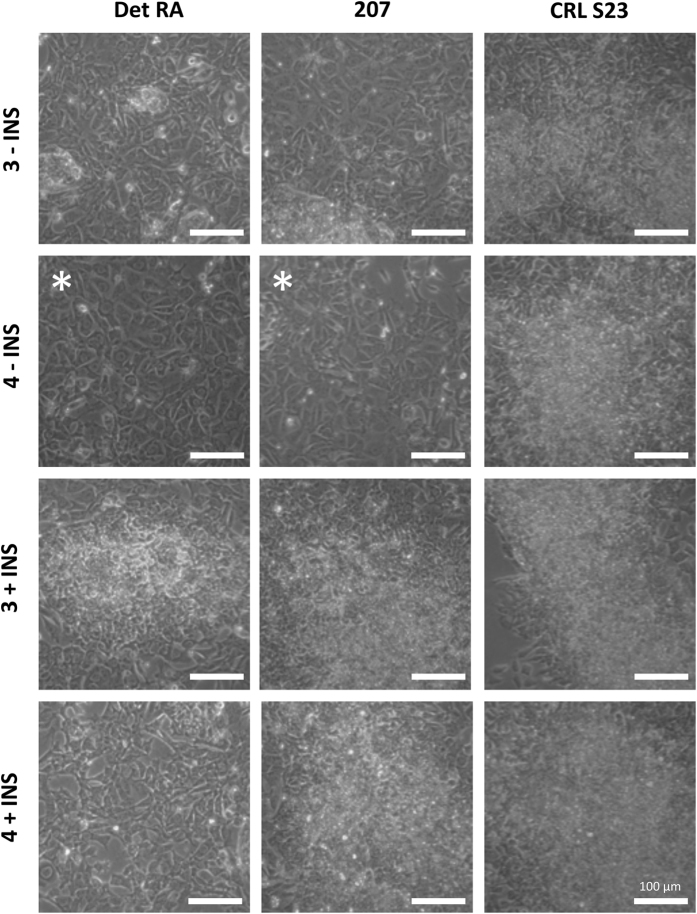
Multi-line EP screen day 2 morphology. Morphology of multiple hPSC lines at day 2 of the EP screen, using 3 and 4 μM CHIR99021 in RPMI supplemented with B27 +/− insulin. The conditions used are indicated to the left side of each row, while the cell line is indicated at the head of each column, −INS = B27 containing no insulin and +INS = B27 containing insulin. The white asterisk indicates the optimal condition for each line. The following hPSC lines were assessed: hiPSC Det RA, hESC 207, and hiPSC CRL S23. Cells were imaged using phase contrast microscopy. See also Supplementary Figs S2 and S3.

**Figure 5:**
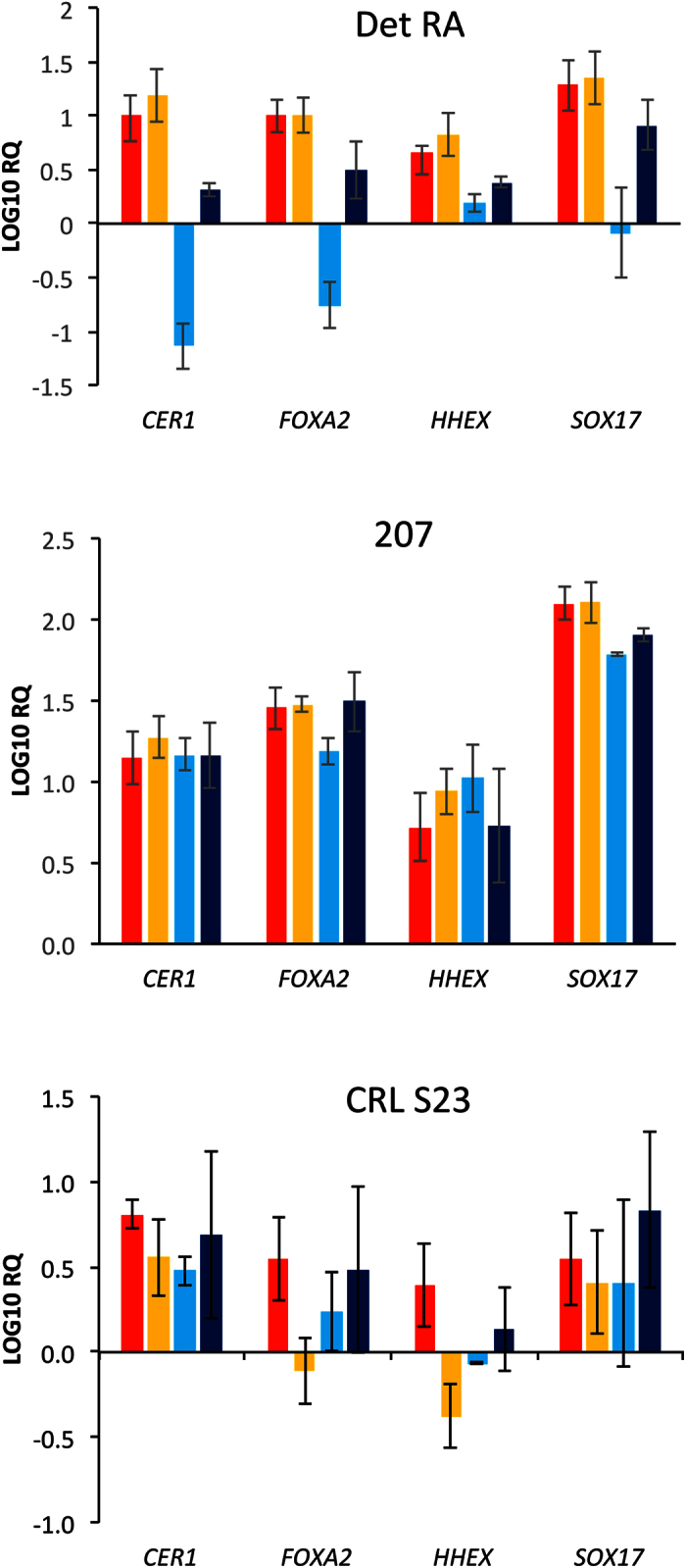
Multi-line EP screen gene expression analysis. The expression of DE specific markers was measured after 48 hours using TaqMan pRT-PCR on the hPSC lines subjected to the EP screen. We analysed a number of DE specific markers: cerberus 1 (*CER1*), forkhead box a2 (*FOXA2*), hematopoietically expressed homeobox (*HHEX*) and SRY (sex determining region Y)-box (*SOX17*). hESC Line: 207. hiPSC Lines: Det RA and CRL S23. The Y-axis represents the LOG10 relative quantification (RQ). All samples were normalized to beta-actin (*ACTB*), and to their respective undifferentiated starting hPSC population. Data is presented as the average of three independent experiments +/− the standard deviation. Red bars = 3 μM CHIR99021/B27 without insulin. Yellow bars = 4 μM CHIR99021/B27 without insulin. Light blue bars = 3 μM CHIR99021/B27 with insulin. Dark blue bars = 4 μM CHIR99021/B27 with insulin. See also Supplementary Fig. S4.

**Figure 6:**
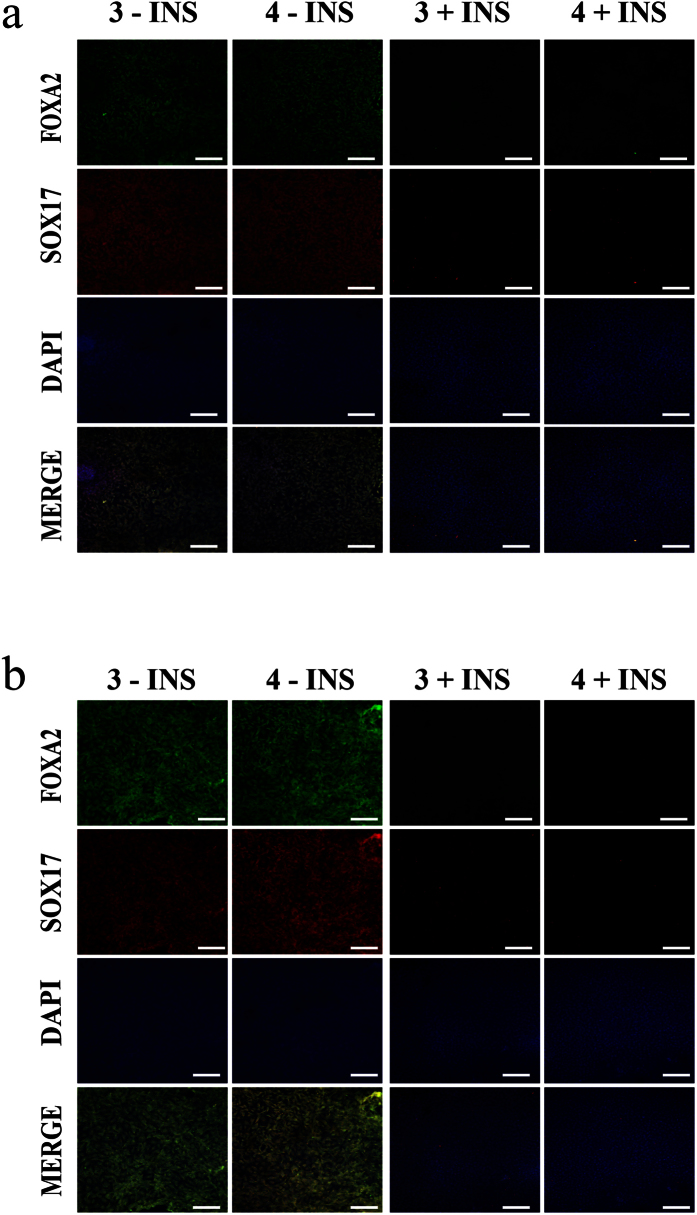
Multi-line EP screen immunohistochemistry. Immunohistochemistry of multiple hPSC lines at day 2 of the EP screen, using 3 and 4 μM CHIR99021 in RPMI supplemented with B27 +/− insulin. The conditions used are indicated on the left side of each row, −INS = B27 containing no insulin and +INS = B27 containing insulin. The following hPSC lines were assessed: (**a**) hiPSC line Det RA, after the above treatment regimes the resulting cells were co-stained for the presence of forkhead box a2 (FOXA2) (green) and SRY (sex determining region Y)-box SOX17 (red), and counterstained with DAPI (blue) and imaged using confocal microscopy. (**b**) hESC 207, after the above treatment regimes the resulting cells were co-stained for the presence of forkhead box a2 (FOXA2) (green) and SRY (sex determining region Y)-box SOX17 (red), and counterstained with DAPI (blue) and imaged using confocal microscopy. Scale bar 100 μM. See also Supplementary Figs S5 and S6.

**Figure 7:**
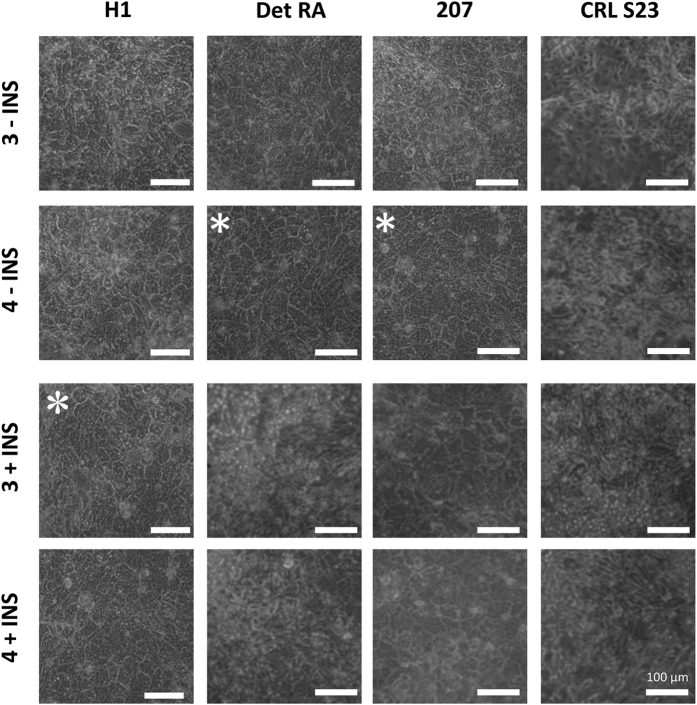
Multi-line EP screen HLC differentiation: Morphology. hPSC lines were differentiated to hepatocyte-like-cells using a small molecule driven differentiation protocol^12^. At the conclusion of the hepatocyte differentiation protocol (day 17), the cells were assessed for the presence of a typical hepatocyte morphology using phase microscopy. The conditions used for definitive endoderm differentiation (EP screen) is indicated to the left of each row, the top line provides the identity of the hPSC line: hESC lines H1 and 207; hIPSC lines Det RA and CRL S23. The best condition is indicated with a white asterisk. Scale bar 100 μM. See also Supplementary Fig. S7.

**Figure 8:**
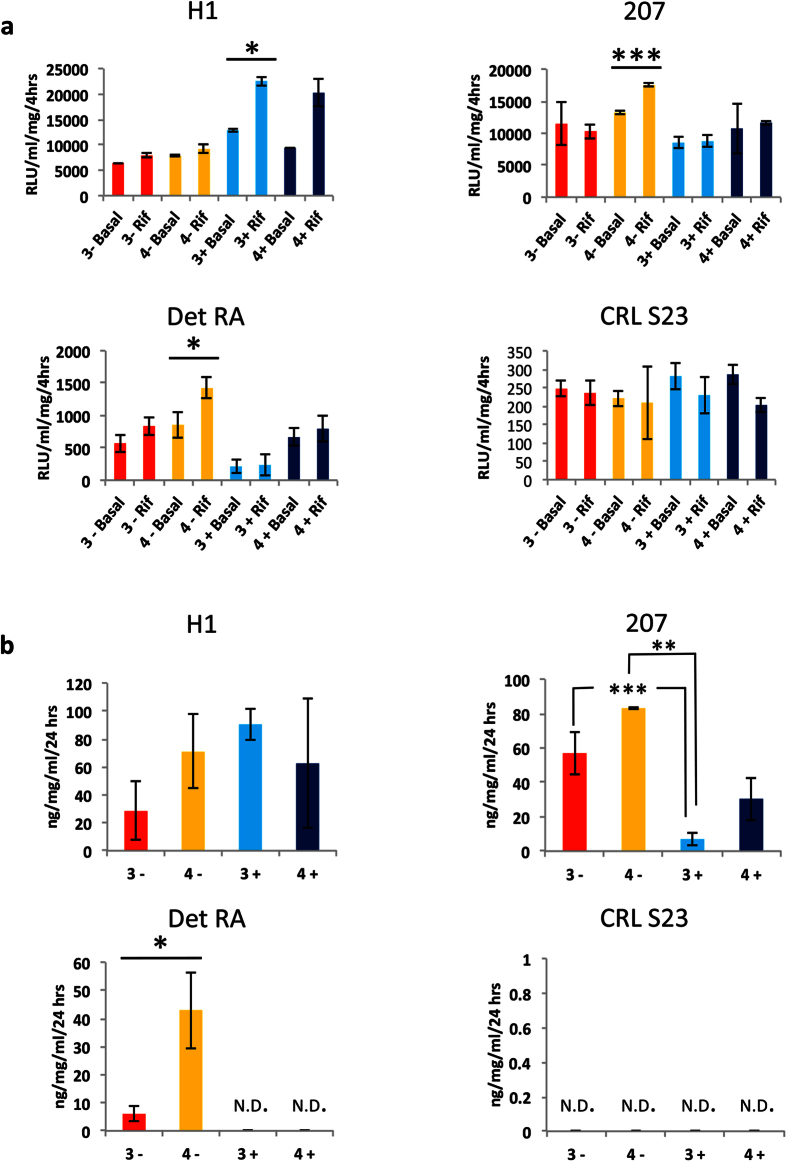
Multi-line EP screen HLC differentiation: Functional assessment of derived hepatocytes. hPSC lines (hESC lines H1 and 207; hiPSC lines Det RA and CRL S23) were differentiated to hepatocytes using a small molecule driven protocol. (**a)** Following the differentiation procedure, cells were assessed for CYP3A4 basal activity and inducibility upon challenge with the well-established inducing agent Rifampicin. Data presented is the average +/− standard deviation; n = 3. All data normalised to total protein. The EP conditions are shown on the X axis of each graph, were the 3 or 4 indicated CHIR99021 concentration, +/− represents the presence or absence of insulin, Basal indicates basal CYP activity and Rif represents induction. The cell line identity is indicated at the head of each graph. (**b)** Hepatocytes were assessed for production and secretion of Albumin by ELISA. All data is presented as the average +/− standard deviation; n = 2. All data normalised to total protein. The EP conditions are shown on the X axis of each graph, were the 3 or 4 indicated CHIR99021 concentration, +/− represents the presence or absence of insulin. The cell line identity is indicated at the head of each graph. Asterisks represent significant differences (2-tailed student´s t test): *p ≤ 0.05; **p ≤ 0.02 and ***p ≤ 0.01.

To provide an alternative strategy that is rapid, cost effective and amenable to high throughput we have developed a 48 hour EP screening strategy based purely on morphology (see Fig. 1 and Supplementary Fig. S1). We clearly demonstrate that the observed morphological changes or lack thereof, correlate with the expected gene expression patterns associated with DE specification i.e. *CER1, FOXA2, HHEX*, and *SOX17.* In addition, we further corroborate these observations with immunohistochemistry against FOXA2 and SOX17. Finally, we have verified that only the lines that pass the EP screen are capable of further differentiation to HLCs. The results of the HLC differentiation correlate to the initial results of the EP screen in two critical aspects: first, only the lines which passed the EP screen are capable of differentiation into HLCs; second, the optimal conditions which were identified during the EP screen for producing DE were the conditions that yielded the most functional and robust hepatic phenotype.

In conclusion, the described EP screening procedure has a number of key benefits, not only does it provide a rapid, morphological readout which can be assessed using common laboratory equipment such as a phase contrast microscope. It importantly provides the user with key information about the optimal conditions to apply to that particular line for further differentiation towards endodermal lineages. Additionally, by combining this EP screening strategy with new technologies such as the Cell Profiler Platform, it will be possible to implement a fully automated, high throughput screening strategy to assess multiple hPSC lines endodermal differentiation capacity^46^.

## Methods

### Human Pluripotent Stem Cell Culture

Human pluripotent stem cells were cultivated on GelTrex (Life Technologies) coated plates, under feeder free conditions using Essential 8 Media (Life Technologies). Cells were passaged when they reached between 80–90% confluency, using 0.5 mM EDTA (Life Technologies).

### Human Pluripotent Stem Cell Lines

In this study we used the following hESCs: H1 (purchased from WiCell)^39^; 207, 360, and 429 obtained from the Karolinska Institute^47^. BJ (CRL-2522), CRL 2097 and Detroit 551 (CCL-110) fibroblasts were obtained from the American Type Culture Collection. For retroviral based hiPSC generation, hOCT4, hSOX2, hKLF4 and hcMYC, retrovirus viral particles were purchased from Vectalys and transduced at an MOI of 5 as previously described by Vallier and colleagues^48^. For non-integrative Sendai virus mediated reprogramming, we used the CytoTune™-iPS 2.0 Sendai Reprogramming Kit (Life Technologies) containing polycistronic Klf4–Oct3/4–Sox2, cMyc, and Klf4, according to the manufactures instructions for feeder free derivation. For both retro- and Sendai virus reprogramming, hiPSC colonies were manually picked into Matrigel (Sigma) coated dishes, cultivated and expanded clonally in Essential 8 Media (Life Technologies). The hiPSC cell lines were verified for the expression of pluripotency related genes by immunofluorescence, FACS, karyotyping and RT-qPCR^11^.

### Endodermal Potential Screen (Definitive Endoderm Screen)

The EP screen was set up in 12 well plate format; the plates were coated with human recombinant vitronectin (Life Technologies) according to manufacturer’s instructions. For immunohistochemistry, coverslips (VWR) were first sterilised by UV treatment for 30 minutes, followed by coating with vitronectin as per manufacturer. hPSC lines were seeded into 12 well plates 1 day prior to initiation of the EP screen. In general, the cells from one well of a 6 well plate (80% confluent) were seeded into 7–9 wells of a 12 well plate. The screen was set up as follows, two concentrations of CHIR99021 (Stemgent - purity of 97% or less) were used: 3 and 4 μM. This was supplemented into either RPMI/B27 with or without insulin (Life Technologies). Cells were cultured for 24 hours in the presence of CHIR99021, followed by 24 hours in either RPMI/B27 with or without insulin. Following this treatment cells were assessed morphologically using phase microscopy (Zeiss) and for the validation the cells were collected for RNA extraction or fixed for immunohistochemistry.

### Hepatocyte Differentiation

The hPSC lines were differentiated to HLCs using a modified version of the previously described protocol^11^. Briefly, cells were passaged using ultra-pure EDTA (Life technologies) and seeded onto coverslips or tissue culture plates previously coated with human recombinant vitronectin (Life Technologies) according to manufacturer instructions. 24 hours after seeding, the media was exchanged to RPMI supplemented with either B27 with or without insulin (Life Technologies) and 3 or 4 μM CHIR99021 (Stemgent). The optimal conditions were determined based on the EP screen described above. The cells were maintained in RPMI/B27 plus CHIR99021 for the first 24 hours, followed by its removal and re-feeding with just RPMI/B27 (with or without insulin) and maintained for a further 24 hours. The media was exchanged for SR/DMSO media for hepatic progenitor specification as described previously^5^,^9^,^11^. The hepatic progenitor specification phase lasted 5 days, with media exchanges every 48 hours. On completion of hepatic progenitor specification (day 7) the media was changed to hepatic induction media, as described previously^11^. Hepatic induction lasted for 9 days, with media exchanges every 48 hours. Upon completion of the hepatic differentiation protocol, the cells were processed for RNA extraction and immunohistochemistry as previously described^11^.

### RNA Isolation, cDNA Synthesis and Reverse Transcription Quantitative Real Time Polymerase Chain Reaction Analysis

Cells were collected for RNA isolation by washing with PBS (Life technologies), scraping the cells into PBS followed by centrifugation at 12 000 × g for 1 minute. Cells were lysed in TRIZOL reagent (Life Technologies). RNA extraction was performed according to the manufacturer’s instructions. RNA was quantified using the NanoDrop ND-1000 Spectrophotometer System (NanoDrop). 500 ng of RNA was converted to cDNA using the High Capacity Reverse Transcription kit (Life Technologies) according to the manufacturer’s instructions. Reverse transcription quantitative real time polymerase chain reaction (RT-qPCR) was performed using TaqMan Reagents (Life Technologies) with the only modification being that the reaction volume was reduced from 20 μl to 15 μl. Probe details are provided in Supplementary Table S1. In all cases 3 biological replicates were analysed using 5 ng of cDNA per reaction. In all cases 3 technical replicates were performed for all samples/genes. For DE, gene expression was normalised to (beta-actin) *ACTB* and against the starting population of pluripotent stem cells for each cell line. For the HLCs, gene expression was normalised to *ACTB* and against DE for each cell line. Data is presented as the average of 3 biological replicates; error bars represent the standard deviation of the biological replicates.

### Immunohistochemistry

After differentiation, cells were washed with PBS (Life technologies) and then fixed with ice-cold methanol (Sigma) for 10 minutes at −20 °C. Cells were then washed with PBS and either processed immediately or stored at 4 °C. Cells were blocked for 3 hours in 10% Normal Goat Serum (Life Technologies), diluted in 0.1% PBS-Tween (Sigma) (PBS-T). Following the blocking step, the cells were washed 2 times with PBS-T and incubated overnight with primary antibodies diluted in 1% Normal Goat Serum/PBS-T. After primary antibody incubation, the cells were washed 4 times with PBS-T and incubated with fluorophore labeled secondary antibodies diluted in PBS for 1 hour at room temperature in the dark. Following secondary antibody incubation, the cells were washed 4 times with PBS-T followed by an additional 4 times with PBS. Coverslips were mounted onto glass microscope slides (Thermo Scientific) using Fluoroshield containing DAPI (Sigma). Antibody details can be found in Supplementary Table S2.

### Quantitative Immunohistochemistry

After differentiation to DE, cells were stained for the presence of SOX17 as described above. After imaging using confocal microscopy, the cells were manually counted. The images were randomised to minimise bias. SOX17 positive and negative cells were counted in 4 fields of view and percentages were then determined. A minimum of 300 cells were counted in each field of view. The data is presented as the average of the 4 fields of view +/− the standard deviation.

### CYP450 Activity

Differentiated HLCs were assessed for CYP3A4 activity and inducibility as follows. On day 20 of the differentiation procedure, the cells were washed 4 times with PBS and then treated with L15 medium with dihexa (without Hydrocortisone 21-hemisuccinate or dexamethasone) supplemented with 25 μM Rifampicin (Sigma). This media was replaced every 24 hours for 72 hours. After 72 hours of treatment, CYP3A4 activity was assessed using the P450-Glo CYP3A4 (Luciferin-PFBE) Cell-Based/Biochemical Assay (Promega) according to manufacturer instructions. CYP3A4 activity was normalised to total protein in each well. Basal activity refers to the measured activity in cells which were not challenged with Rifampicin.

### Albumin Production

Albumin production was assessed on terminally differentiated HLCs as follows. Cells were incubated for 24 hours with fresh L15 medium containing dexamethasone and Dihexa. After the incubation, the media was collected, flash frozen on dry ice and stored at −80 °C until further analysis. Albumin in the supernatants was assessed using the human specific ELISA kit (Alpha Diagnostics). All data was normalised to total protein in the well. Data is presented as the mean of two experiments +/− the standard deviation.

### Protein Quantification

Protein content of the differentiated cells was assessed as follows. Cells were lysed in TPER buffer (Thermo Scientific) and passed through a needle several times to fully lyse cells. The protein content of the resulting lysates was then quantified using the BCA Assay Kit (Pierce) and analysed on an absorbance plate reader (Tecan).

### Periodic Acid-Schiff Staining

Differentiated HLCs were assessed for glycogen storage using the Periodic Acid-Schiff Staining Kit (Sigma) according to manufacturer instructions.

### Imaging

Phase contrast imaging was performed on a PrimoVert microscope (Zeiss) and images were acquired using ZEN software from Zeiss. Confocal imaging was performed on a Zeiss LSM700 confocal microscope and images were acquired using ZEN software from Zeiss.

### Statistics

Statistical significance was determined using two tailed student’s t test, with p ≤ 0.05 determined to be significant.

## Additional Information

**How to cite this article**: Siller, R. *et al*. Development of a rapid screen for the endodermal differentiation potential of human pluripotent stem cell lines. *Sci. Rep.* **6**, 37178; doi: 10.1038/srep37178 (2016).

**Publisher’s note:** Springer Nature remains neutral with regard to jurisdictional claims in published maps and institutional affiliations.

## Supplementary Material

Supplementary Information
