# Supplementary material for: Development of a rapid screen for the endodermal differentiation potential of human pluripotent stem cell lines
Source: Sci Rep. 2016 Nov 22;6:37178. doi: 10.1038/srep37178 (PMC5118706; doi:10.1038/srep37178)

Title: Development of a rapid screen for the endodermal differentiation potential of human pluripotent stem cell lines.

Richard Siller[1], Elena Naumovska[1], Santosh Mathapati[1], Max Lycke[1], Sebastian Greenhough[1] and Gareth J Sullivan[1, 2, 3]*

[1] Department of Biochemistry, Institute of Basic Medical Sciences, Faculty of Medicine, University of Oslo, PO Box 1112 Blindern, 0317 Oslo, Norway.

[2] Norwegian Center for Stem Cell Research, PO Box 1112 Blindern, 0317 Oslo, Norway.

[3] Institute of Immunology, Oslo University Hospital-Rikshospitalet, PO Box 4950 Nydalen, Oslo 0424, Norway.

*Correspondence to: gareth.sullivan@medisin.uio.no

Tel nos. +47 22851415

Supplementary Figure S1. EP screen schematic.

a. Schematic detailing the timing for the endodermal potential (EP) screen. **b.** Diagram illustrating the endodermal potential screen set up, in terms of CHIR99021 (CHIR) concentrations (3 – 4μM) and presence or absence of Insulin in the B27 supplement (+/ -). See also Fig. 1.

Supplementary Figure S2. Multi-line EP screen day 2 morphology.

Morphology of multiple hPSC lines at day 2 of the EP screen, using 3 and 4 μM CHIR99021 in RPMI supplemented with B27 +/- insulin. The conditions used are indicated to the left side of each row, while the cell line is indicated at the head of each column, -INS = B27 containing no insulin and +INS = B27 containing insulin. The white asterisk indicates the optimal condition for each line. The following hPSC lines were assessed: hESC lines 360 and 429; and hiPSC line BJ S1. Cells were imaged using phase contrast microscopy. Scale bar 100 μM. See also Figs. 2 and 4 and Supplementary Fig. S3 online.

Supplementary Figure S3. Multiline EP screen day 2 morphology.

Morphology of multiple hPSC lines at day 2 of the EP screen, using 3 and 4 μM CHIR99021 in RPMI supplemented with B27 +/- insulin. The conditions used are indicated to the left side of each row, while the cell line is indicated at the head of each column, -INS = B27 containing no insulin and +INS = B27 containing insulin. The white asterisk indicates the optimal condition for each line. The following hPSC lines were assessed: hiPSC lines Det RB, Det RC and CRL R5. Cells were imaged using phase contrast microscopy. Scale bar 100 μM. See also Figs. 2 and 4 and Supplementary Fig. S2 online.

Supplementary Figure S4. Multiline EP screen day 2 gene expression analysis.

The expression of DE specific markers was measured after 48 hours using TaqMan pRT-PCR on the hPSC lines subjected to the EP screen. We analysed a number of DE specific markers:

cerberus 1 (CER1), forkhead box a2 (FOXA2), hematopoietically expressed homeobox (HHEX) and SRY (sex determining region Y)-box (SOX17). hESC line: 360 and 429; hiPSC lines: BJ S1, Det RB, Det RC, BJ S1 and CRL S23. The Y-axis represents the LOG10 relative quantification (RQ). All samples were normalized to beta-actin (ACTB), and to their respective undifferentiated starting hPSC population. Data is presented as the average of three independent experiments +/- the standard deviation. Red bars = 3 µM CHIR99021 / B27 without insulin. Yellow bars = 4 µM CHIR99021 / B27 without insulin. Light blue bars = 3 µM CHIR99021 / B27 with insulin. Dark blue bars = 4 µM CHIR99021 / B27 with insulin. See also Fig. 5.

Supplementary Figure S5. Multiline EP screen day 2 immunohistochemistry. Immunohistochemistry of multiple hPSC lines at day 2 of the EP screen, using 3 and 4 µM CHIR99021 in RPMI supplemented with B27 +/- insulin. The conditions used are indicated on the left side of each row, -INS = B27 containing no insulin and +INS = B27 containing insulin. The line identity is indicated at the top of each column, the following hPSC lines were assessed: hESC lines 360 and 429; hiPSC line BJ S1. After the above treatment regimes the resulting cells were co-stained for the presence of forkhead box a2 (FOXA2) (green) and SRY (sex determining region Y)-box 17 (SOX17) (red), and counterstained with DAPI (blue) and imaged using confocal microscopy. Scale bar 100 µM. See also Figure 6 and Supplementary Fig 6 online.

Supplementary Figure S6. Multiline EP screen day 2 immunohistochemistry. Immunohistochemistry of multiple hPSC lines at day 2 of the EP screen, using 3 and 4 µM CHIR99021 in RPMI supplemented with B27 +/- insulin. The conditions used are at the left side of each row, -INS = B27 containing no insulin and +INS = B27 containing insulin. The line identity is indicated at the top of each column, the following hPSC lines were assessed:

hiPSC lines Det RB, Det RC and CRL R5 After the above treatment regimes the resulting cells were co-stained for the presence of forkhead box a2 (FOXA2) (green) and SRY (sex determining region Y)-box SOX17 (red), and counterstained with DAPI (blue) and imaged using confocal microscopy. Scale bar 100 μM. See also Figure 5 and Supplementary Fig. 5 online.

Supplementary Figure S7. Multiline EP screen HLC differentiation: Glycogen storage. Multiple hPSC lines were differentiated to hepatocyte like cells using a small molecule driven protocol[12]. At the end of the differentiation the cells were assessed for their ability to store glycogen via Periodic acid–Schiff (PAS) staining. Cells were observed and imaged using phase contrast microscopy. The conditions used for definitive endoderm induction are indicated to the left of each row. The cell line is indicated at the top of each column. hPSC lines assessed were: hESC line H1 and 207; hiPSC lines Det RA and CRL S23. Scale bar 100 μM. See also Fig. 8.

Supplementary Figure S8. Quantitative immunohistochemistry for SOX17.
Cells were differentiated to definitive endoderm (day 2 of the EP screen) and stained for the presence of SRY (sex determining region Y)-box 17 (SOX17) (red), and counterstained with DAPI (blue) and imaged using confocal microscopy. The SOX17 positive cells were counted and percentages were determined based on counterstaining of DAPI. 4 fields of view with a minimum of 300 cells each were counted for each line and each differentiation condition. Data presented as the average +/- standard deviation. For submission Asterisks represent significant differences (2-tailed student´s t test): *$p \leq 0.02$; **$p \leq 0.01$ and ***$p \leq 0.005$.

Supplementary Table S1. TaqMan RT-qPCR Probe Details.

Gene	Catalogue Number (Life Technologies)	Reference
CER1	4331182	Hs00193796_m1
FOXA2	4331182	Hs00232764_m1
GATA4	4331182	Hs00171403_m1
GSC	4331182	Hs00906630_g1
HHEX	4331182	Hs00242160_m1
MIXL1	4331182	Hs00430824_g1
NODAL	4331182	Hs00415443_m1
POU5F1	4331182	Hs00999634_gH
SOX17	4331182	Hs00751752_s1
SOX7	4331182	Hs00846731_s1
T	4331182	Hs00610080_m1
ACTB	4352935E	N/A

Supplementary Table S2. Antibody Details.

Target	Supplier	Catalogue Number	Species	Dilution
FOXA2	AbCam	ab40874	Rabbit	1:1000
SOX17	AbCam	ab84990	Mouse	1:100
Alexaflour 488 anti rabbit	Life Technologies	A21206	Donkey	1:400
Alexafluor 488 anti mouse	Life Technologies	A11059	Rabbit	1:400
Alexafluor 594 anti mouse	Life Technologies	A11005	Goat	1:400

Supplementary Figure 1. SULLIVAN.

a

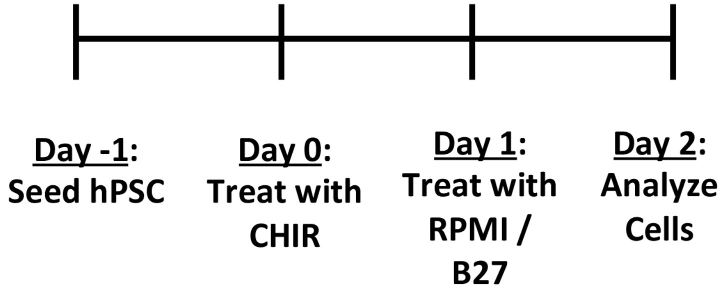

b

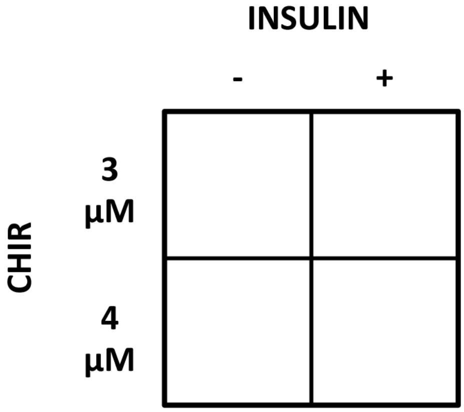

Supplementary Figure 2. SULLIVAN

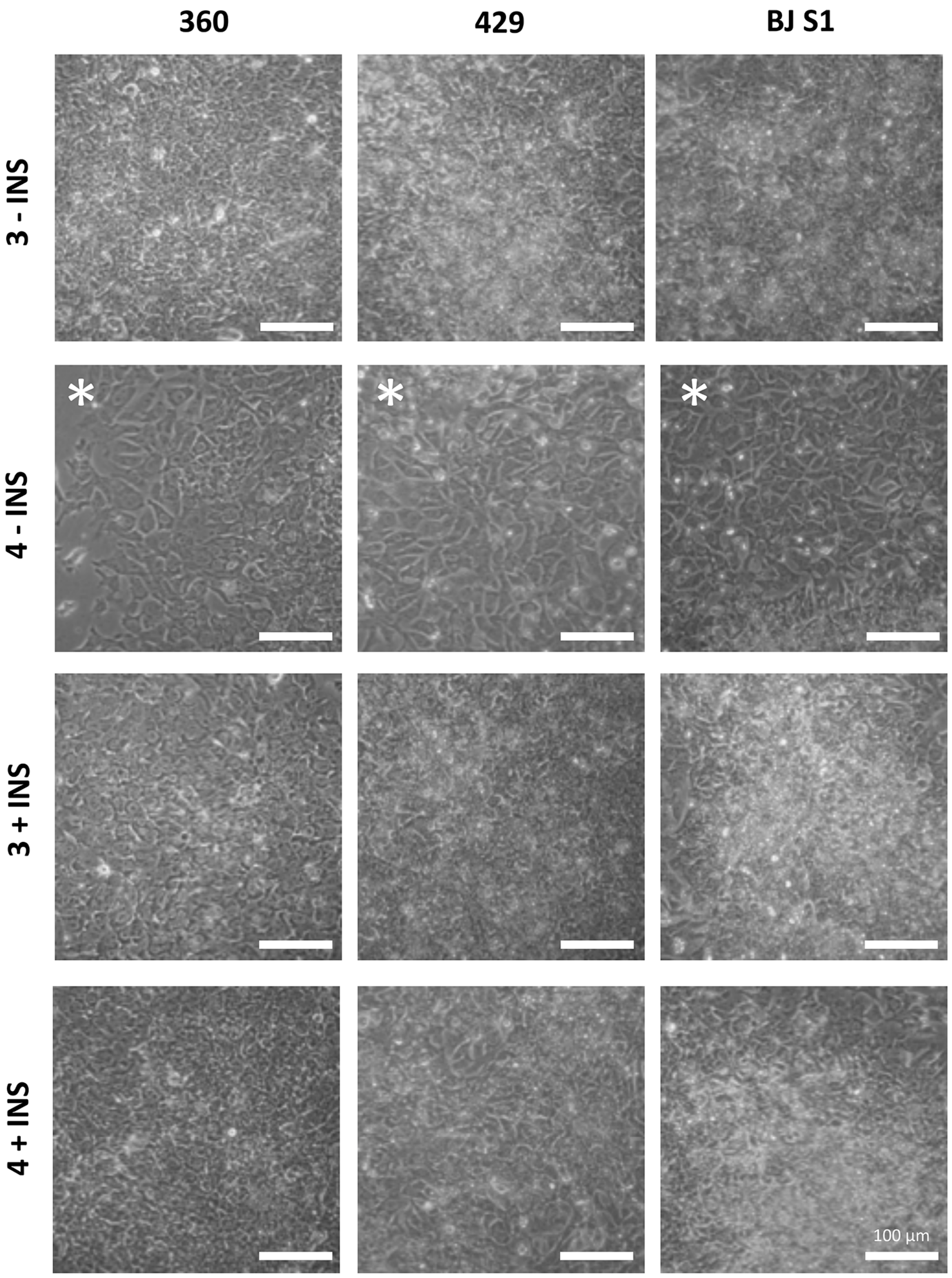

Supplementary Figure 3. SULLIVAN

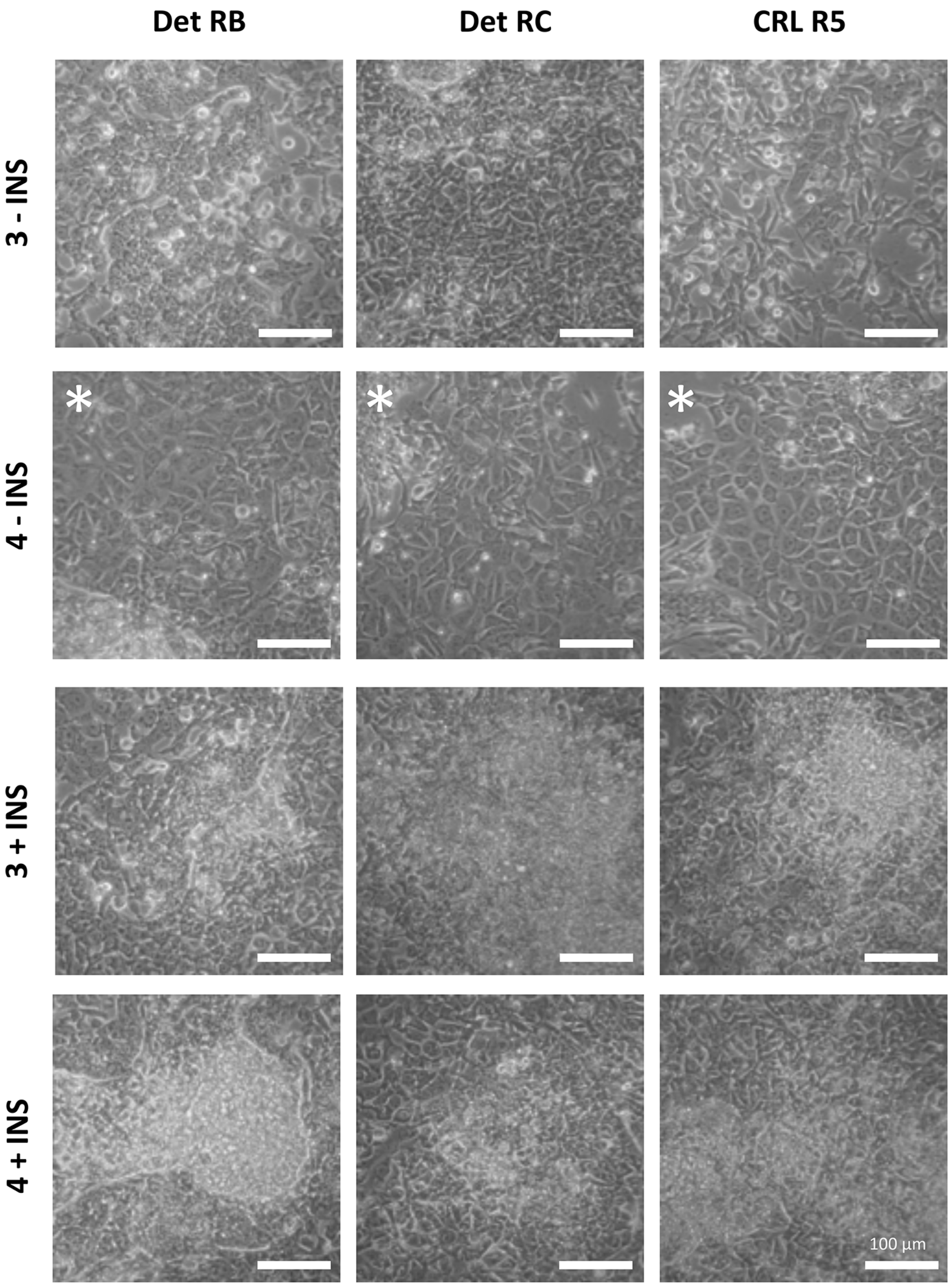

Supplementary Figure 4. SULLIVAN

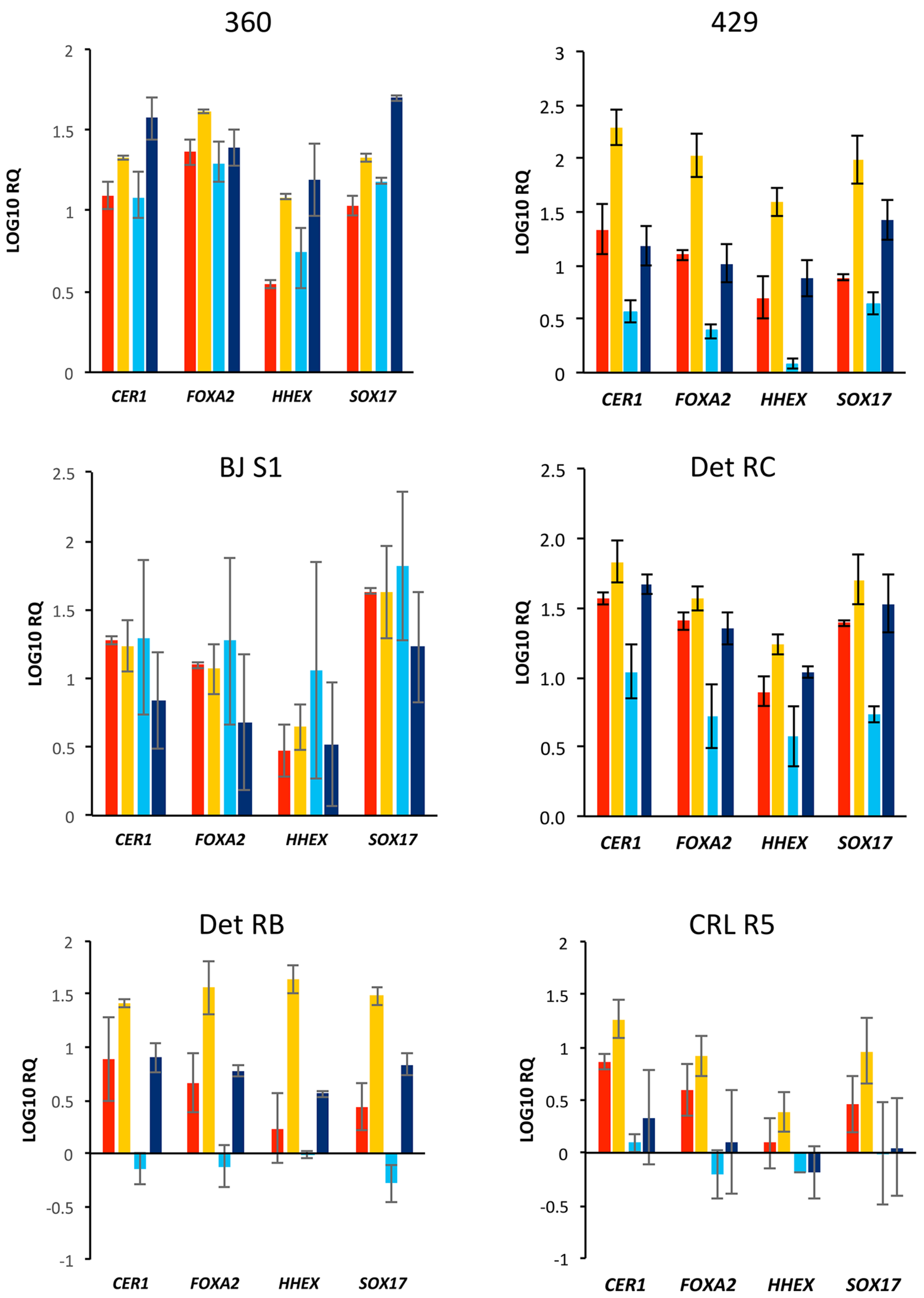

Supplementary Figure 5. Sullivan new

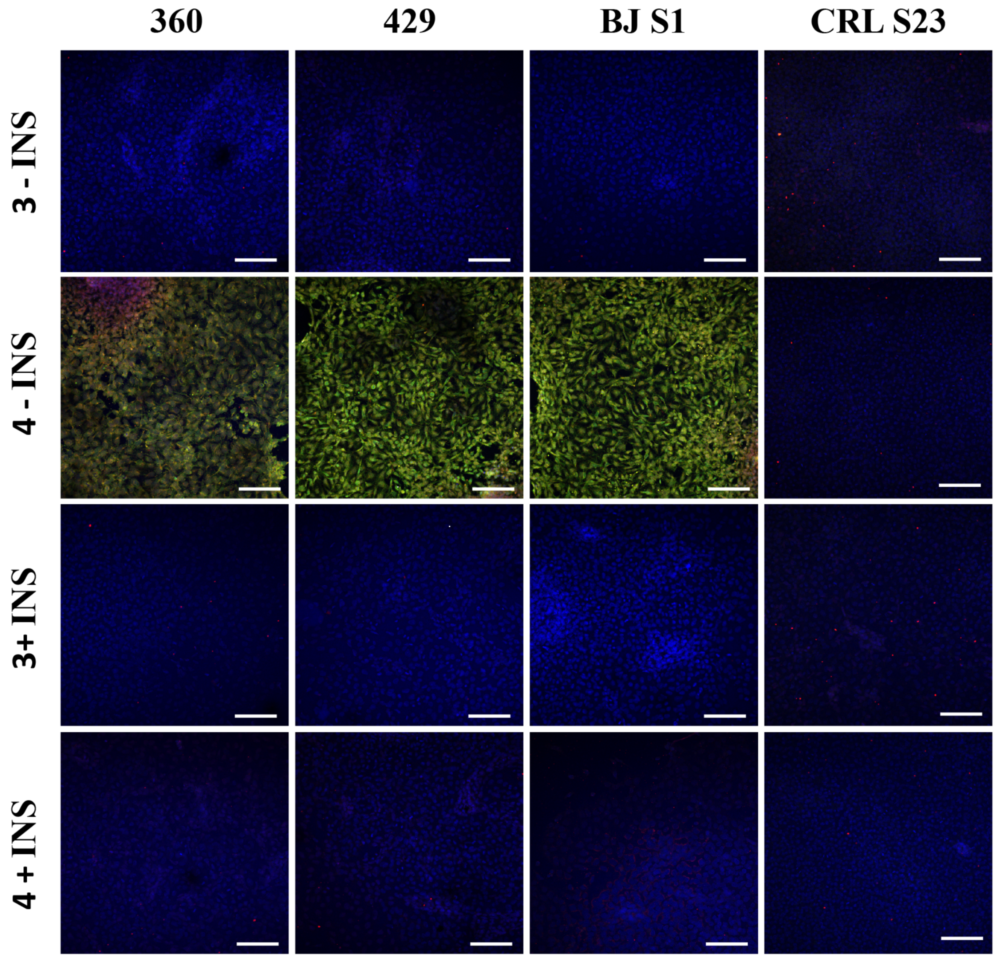

Supplementary Figure 6 new. SULLIVAN

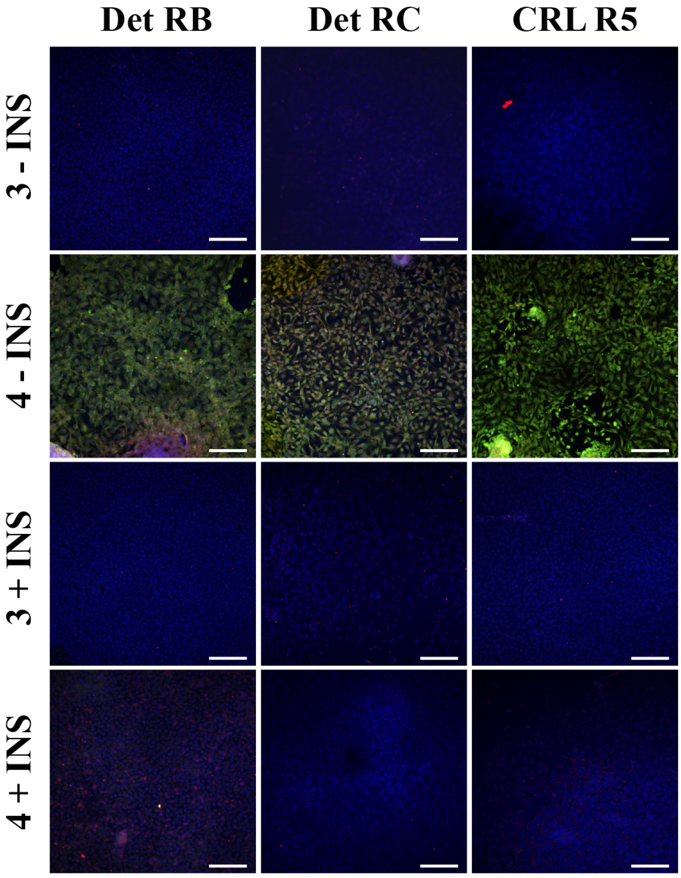

Supplementary Figure 7. SULLIVAN.

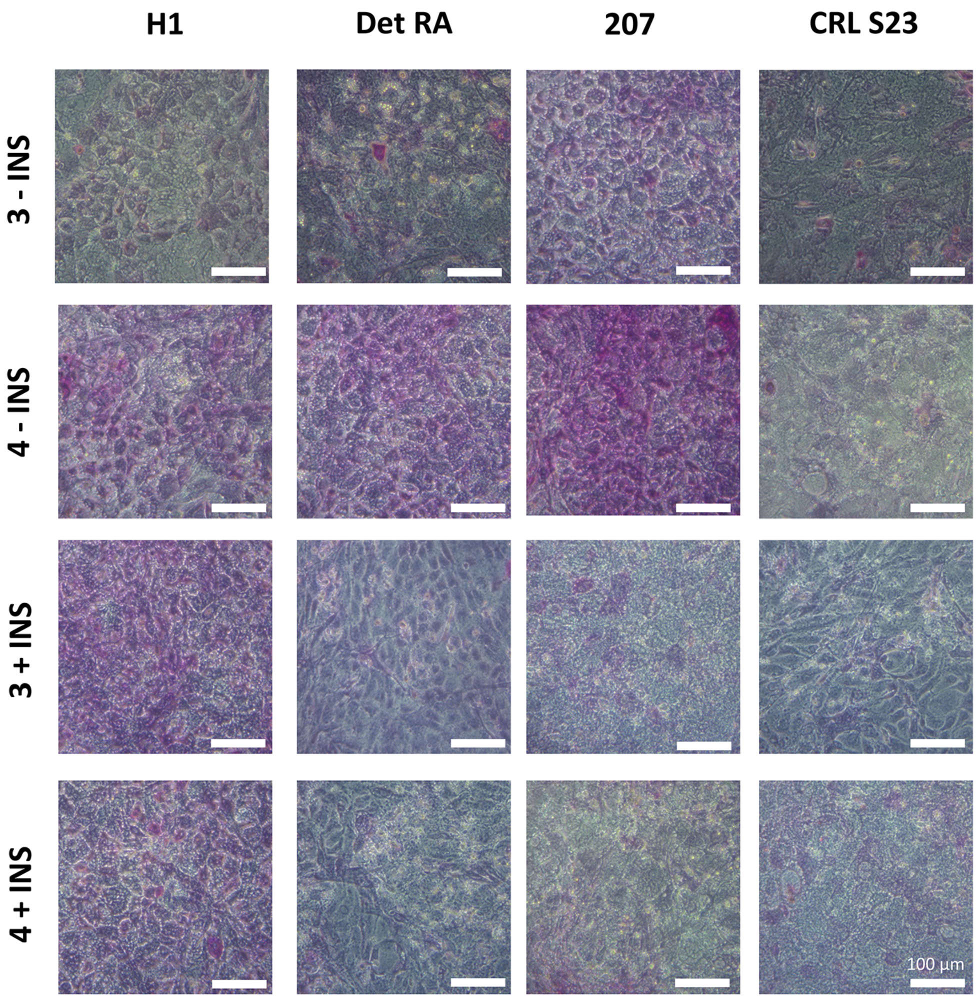

Supplementary Figure 8. SULLIVAN

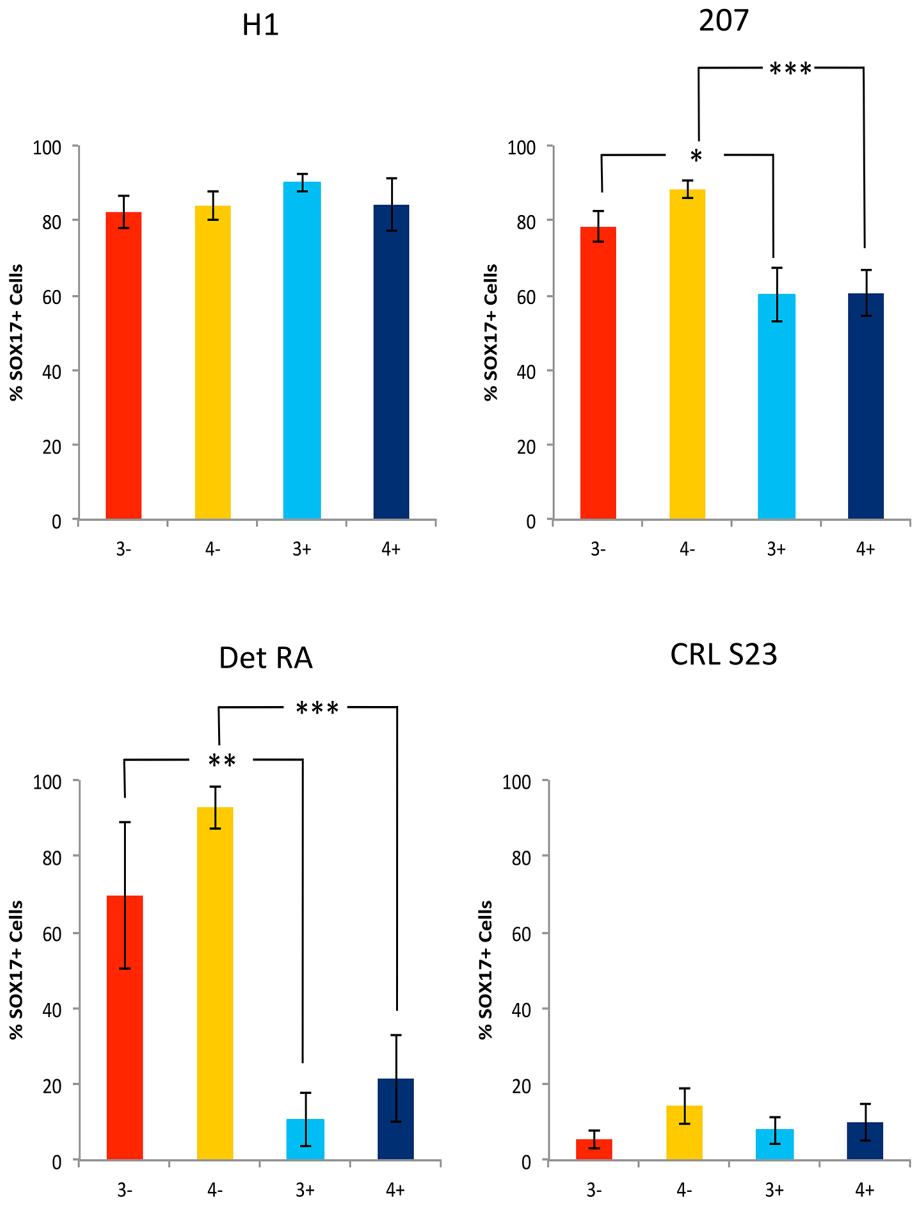

Supplement: Supplementary Information [file srep37178-s1.pdf]
